# Clustered event related spectral perturbation (ERSP) feature in right hand motor imagery classification

**DOI:** 10.3389/fnins.2022.867480

**Published:** 2022-08-16

**Authors:** Zhongjie Zhang, Yasuharu Koike

**Affiliations:** Institute of Innovative Research, Tokyo Institute of Technology, Yokohama, Japan

**Keywords:** brain-computer interface (BCI), motor imagery (MI), event related spectral perturbation (ERSP), right hand, same limb, clustered features

## Abstract

A technology that allows humans to interact with machines more directly and efficiently would be desirable. Research on brain-computer interfaces (BCIs) provides the possibility for computers to understand human thoughts in a straightforward manner thereby facilitating communication. As a branch of BCI research, motor imagery (MI) techniques analyze the brain signals and help people in many aspects such as rehabilitation, clinical applications, entertainment, and system controlling. In this study, an imagery experiment consisting of four kinds of right-hand movements (gripping, opening, pronation, and supination) was designed. Then a novel feature, namely, clustered feature was proposed based on the event-related spectral perturbation (ERSP) calculated from the EEG signal. Based on the selected features, two classical classifiers (support vector machine and linear discriminant classifier) were trained, achieving an acceptable accurate result (80%, on average).

## 1. Introduction

In recent decades, the development of the brain-computer interface (BCI) has provided novel and intuitive interactions that make our lives more convenient. In medicine, BCI helps stroke patients with rehabilitation, the disabled to control prosthetic limbs, and amyotrophic lateral sclerosis (ALS) patients to communicate with others. In addition, various BCI studies have been conducted on remote robot control, emotion analysis, and entertainment equipment.

The implementation of these applications is inseparable from the brain activities signal measurement. There are different measurement techniques, such as functional magnetic resonance imaging (fMRI), magnetoencephalography (MEG), electrocorticography (ECoG), and electroencephalogram (EEG) for recording the signals representing brain activity. ECOG is an invasive method that records high-quality signals reflecting delicate brain activities, such as finger movement (Yoshimura et al., 2017). However, there is a risk associated with ECoG because the electrodes or probes are set on the exposed surface of the cortex. Therefore, research on signals from non-invasive methods, such as fMRI, MEG, and EEG signals has become more popular. MEG and fMRI provide detailed brain activity analysis with high spatial resolution (Ramadan and Vasilakos, 2017) although the temporal resolution is low because of the measurement technique. Meanwhile, the devices used to acquire fMRI and MEG are bulky, making it inconvenient to move or carry. In contrast, EEG applications have characteristics opposite to those of fMRI and MEG. The advantages of high temporal resolution, portability, and safety make EEG applicable to various scenarios, regardless of indoor or outdoor environment.

EEG signal-based research, such as sentiment analysis, motor control, attention detection, and motor imagery have been applied in many different fields to help unravel the complexity of brain activity. Analyzing the EEG signal provides insight into the activities of the brain and control devices. Motor imagery (MI) research plays a significant role in bridging this gap for people and devices, such as prosthetic limbs, robotic assistance systems, and rehabilitation equipment (Miladinović et al., 2020). As a result, there are a number of EEG signal-based motor imagery studies, such as the classification of the right vs. left hand or hand vs. feet movement (Pfurtscheller et al., 2006). However, the analysis of the same limb motor imagery is still challenging because of the complexity of the brain activity and low discrimination of the EEG signal (Srinivasan, 1999; Corley and Huang, 2018). Thus, the main contributions of this study are as follows:

1. A motor imagery experiment was designed to record the EEG signal of the right-hand imagery movement (grip, opening, pronation, and supination). Through a visual stimulus, the subjects could obtain a detailed hint on how to perform the motor imagery task.
2. A novel feature calculation method was proposed to enhance brain oscillatory activities in the frequency domain. To improve the classifier performance, clustered event relative spectral perturbation feature was proposed as input.
3. A comparative study on the feature performance of different baseline. Comparing the baseline before observation period and imagery period, we obtain an acceptable accuracy from each subject on a small size dataset. This result provides a possibility that the strategy can be changed as an online real-time classification after the quick configuration of collecting the subject's signal.

## 2. Previous and relative research

In contrast to motor movement, in which the EEG signal is recorded while the subject is performing the actual action, motor imagery is a mental process that rehearses or simulates a given action while the subject does not move his/her body or limbs (Decety and Ingvar, 1990). Therefore, motor imagery bridges the gap in communication between people with disabilities, such as those wearing prostheses and devices, and computers (Kappes and Morewedge, 2016). Moreover, healthy people can also enjoy the benefits of MI research.

Physiological arguments indicate that motor imagery is strongly related to brain activity in the μ (8–12 Hz) and β (16–24 Hz) bands (da Silva, 1991). By analyzing the signals in these two bands, various MI applications have been developed as novel interfaces between humans and computers, such as prostheses (Elstob and Secco, 2016), exoskeletons (Rodríguez-Ugarte et al., 2018), drones (Kos' Myna et al., 2014), and robotic arms (Meng et al., 2016).

Researchers have attempted to improve the analysis accuracy of high sensitivity EEG signals in various aspects through preprocessing, feature extraction, and classifier design. Preprocessing methods consisting of spatial filters, such as the common average reference (CAR; McFarland et al., 1997), classical Laplacian method (Hjorth, 1975), spherical spline Laplacian (Perrin et al., 1989), and adaptive methods (Togha et al., 2019) have been used to improve the signal-to-noise ratio (SNR) of the EEG signal and suppress artifact interference.

In addition to the research on signal preprocessing, the development of algorithms for feature extraction, based on event-related desynchronization (ERD; Pfurtscheller and Aranibar, 1977), event-related synchronization (ERS; Pfurtscheller, 1992), event-related potential (ERP; Luck, 2014) have also promoted MI research (Bashashati et al., 2007; Ramadan and Vasilakos, 2017). Feature extraction methods, such as wavelet transformation (WT; Adeli et al., 2003; Hsu and Sun, 2009) and Fourier transformation (Akin, 2002; Polat and Güneş, 2007) are effective in MI classification. Besides, common spatial pattern (CSP; Wang et al., 2006) method, which extremizes the variance of two-class signals, is widely applied in MI research. Methods based on CSP, such as filter bank common spatial pattern (FBCSP; Abbas and Khan, 2018; Das et al., 2020) also perform very well in MI classification.

Designing an efficient classifier is an important part of MI research. Classic classifiers including linear discriminant analysis (LDA; Tariq et al., 2019), support vector machine (SVM; Suwannarat et al., 2018), Naïve Bayesian classifier (NBC; Ang et al., 2012), classification and regression tree (CART; Bentlemsan et al., 2014), and k-nearest neighbor (kNN; Lotte et al., 2007), are widespread in various motor imagery tasks. With the development of the computing power, the neural network such as convolutional neural network (Lawhern et al., 2018) and deep learning methods have attracted increasing attention in recent years (Schirrmeister et al., 2017; Ma et al., 2019). In this study, the performance of SVM and LDA are compared in the right hand motor imagery classification.

## 3. Experiment and methodology

### 3.1. Experiment design

Nine healthy subjects (seven males and two females), aged 23–31 years (mean, 26.1; SD, 2.7) participated in the motor imagery experiment. All of them were right-handed. None of the subjects had any physical or psychological diseases. Studies involving human participants were reviewed and approved by the ethics committee of the Tokyo Institute of Technology (ethics number: 2019001). The patients provided written informed consent to participate in the study.

**Figure 1:**
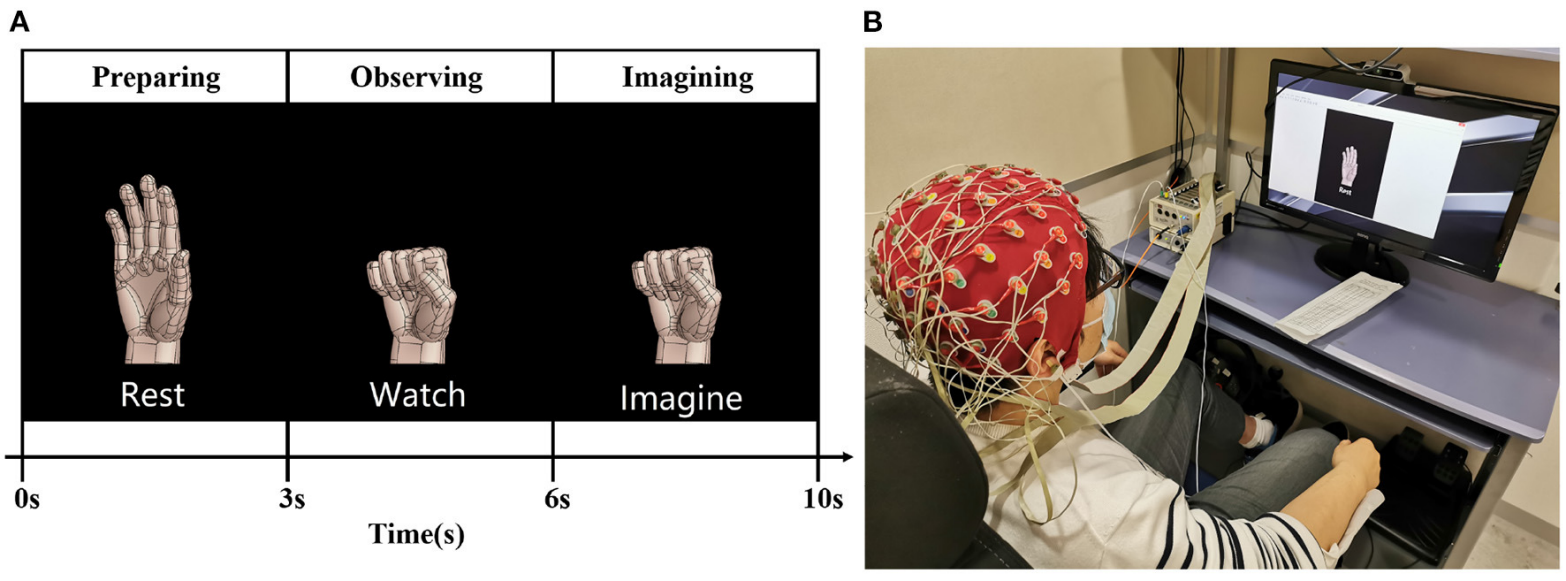
The experimental design of right hand motor imagery. **(A)** Illustrates the grip trial of right-hand motor imagery. **(B)** Shows that the subject adjusted his position and relaxed during the rest period.

The EEG signal was recorded in a soundproof room. The subjects placed their forearm on the armrest of an armchair in a comfortable sitting posture. In front of the subjects, there was a 27” monitor displaying pictures to guide the subject through experimental instructions. One experimental trial was divided into three parts, as shown in Figure 1A.

1. **Preparation period:** In this period lasting 3 s, the subjects could adjust their sitting posture and feel relaxed to prepare for the instructions.
2. **Observation period:** In this period lasting 3 s, the subjects learned the right hand motor imagery from the figure. As visual stimulus, the figure helps the subject imagine a more detailed right hand movement (Neuper et al., 2005).
3. **Imagination period:** In this period lasting 4 s, the subjects imagined the right hand movement (opening, gripping, pronation, and supination) as shown on the monitor while trying to avoid eye and body movement.

The experiment was repeated 80 times for each participant. A 64-channel-scalp sensor (BIOSEMI Active Two, sampling at 2,048 Hz) was used to record the EEG signal, as shown in Figure 1.

### 3.2. Preprocessing

**Figure 2:**
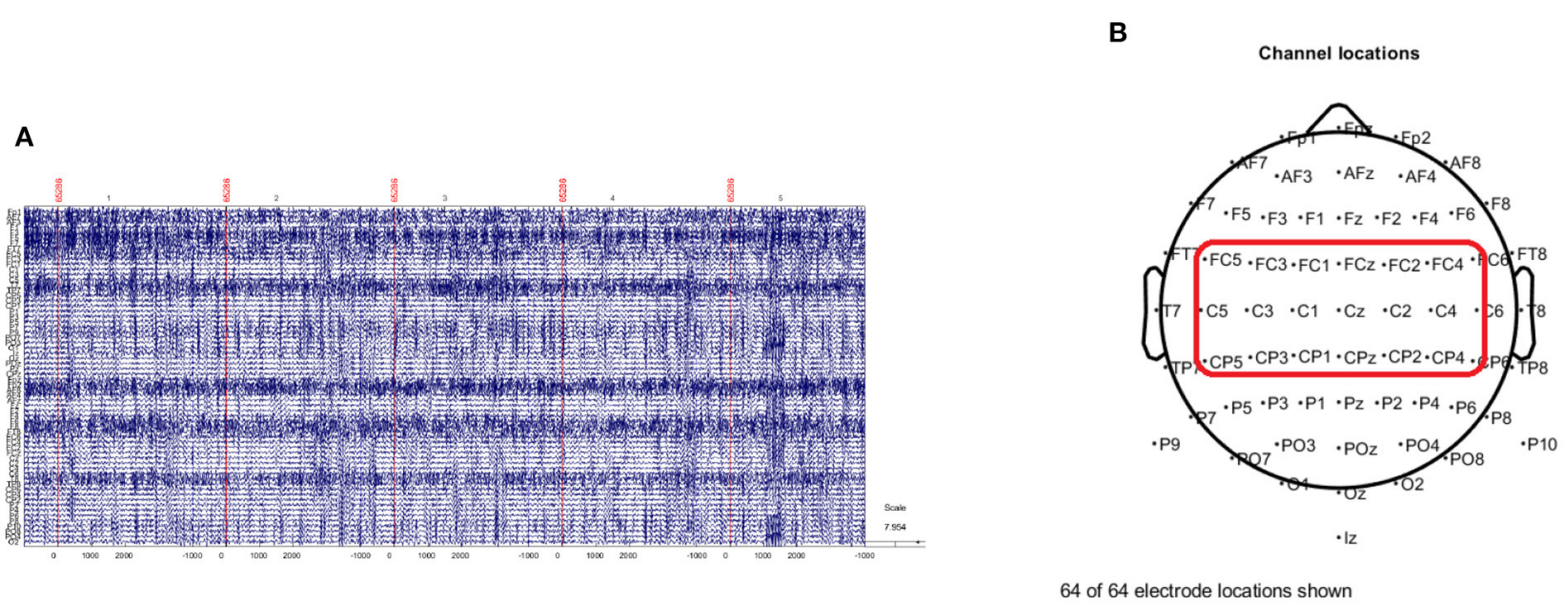
The preprocessing of EEG signal. **(A)** Illustrates the EEG imagery epochs extracted from preprocessed (band pass filter and common average reference) EEG signal. **(B)** Shows the standard 10–20 electrode layout of the 64-channel EEG sensor. Twenty-one channels in the red rectangular area are selected for further processing.

The first stage in preprocessing is to improve the quality of the EEG signal. A Hamming-windowed FIR filter is utilized to acquire the 6–30 Hz EEG data, including the μ (8–12 Hz) and β (16–24 Hz) bands. The common average reference, namely CAR, is a popular and efficient method for enhancing SNR, and is expressed as follows:


(1) $$\begin{array}{r} v_{i}^{\text{CAR}}=v_{i}-\frac{1}{N}\overset{N}{\underset{j=1}{\sum }}v_{j} \end{array}$$

where *v*_*i*_ is the value of the *i*th electrode and *N* is the number of electrodes. By subtracting the average value from all electrode values in large areas of the scalp, CAR emphasizes local activity in the EEG data, such as motor imagery activity in the μ and β bands. As shown in Figure 2A, CAR removes artifacts, such as eye movement and electromyography (EMG), and decreases the correlation between EEG channels (McFarland et al., 1997). Based on the result from previous research and clinical report (Pfurtscheller and Neuper, 1997), human motor movement and motor imagery originate from the motor cortex of the brain. Therefore, 21 channels which cover the motor cortex are selected for the further analysis.

### 3.3. Feature extraction

#### 3.3.1. Event related spectral perturbation (ERSP)

Event related desynchronization and synchronization (ERD/S), which are brain oscillatory activities in specific frequency bands extracted from the EEG signal, are widely utilized in motor imagery research (Pfurtscheller and Aranibar, 1977; Pfurtscheller, 1992). To quantify and generalize ERD/S, the concept of event-related spectral perturbation (ERSP) (Grandchamp and Delorme, 2011) is introduced. ERSP describes the relative changes in the EEG amplitude spectrum of similar events in the experimental trials, and is computed using a divisive baseline and discrete sliding window. The event related spectrum for a single trial can be estimated as


(2) $$\begin{array}{r} P_{i}=\frac{|F_{i}\left(f,t\right){|}^{2}}{\mu_{B}\left(f,i\right)} \end{array}$$

where *F*_*i*_(*f, t*) is the spectral estimate at frequency *f* and time point *t* of the *i*th trial. We apply Fast Fourier Transform (FFT) by using a Hanning window of 512 sample length and 75% overlapping of the window length to assume the spectral estimate. μ_*B*_(*f, i*) is the mean baseline spectral estimate for trial k at frequency f and defined as


(3) $$\begin{array}{r} \mu_{B}\left(f,i\right)=\frac{1}{m}\underset{t^{\prime }\in B}{\sum }|F_{i}\left(f,t^{\prime }\right){|}^{2} \end{array}$$

where *B* is the ensemble of time points in the baseline period and *m* is the total number of time points in the baseline period. The ERSP estimate of one single trial can calculate as


(4) $$\begin{array}{r} ERSP_{single}=10log_{10}\left(P_{i}\right) \end{array}$$

**Figure 3:**
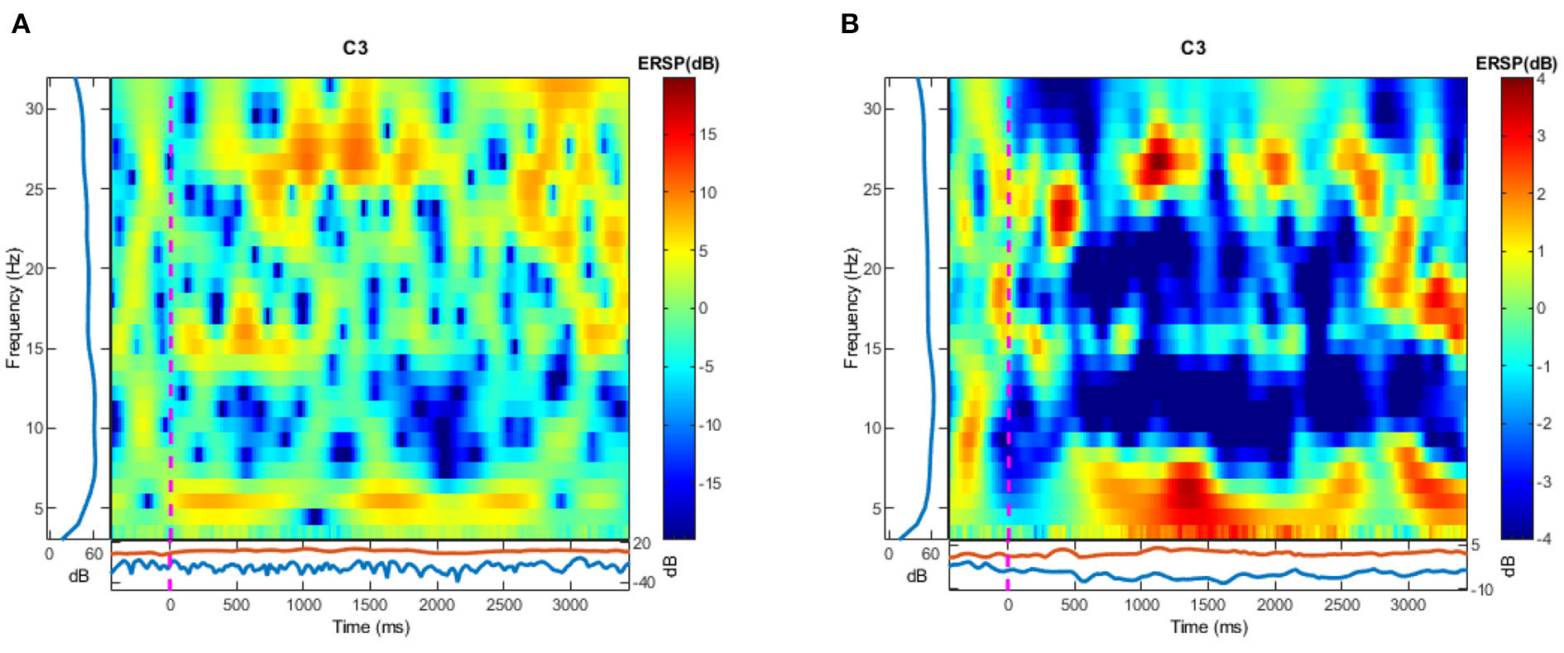
The visualization of ERSP calculation in C3 channel. **(A)** Is the result calculated by one trial and **(B)** is calculated by 10 trials picked randomly from all trials. Blue areas show the ERD while the red areas show the ERS.

Considering the EEG signal is a non-stationary signal with a high degree of variability, even if the signal is from the same subject, it is necessary to reduce the signal's randomness. By averaging the values of ERSP in subjects, we can not only obtain a stationary signal feature, but also can observe the activities of ERS and ERD directly from the visualization as shown in Figure 3. The mean event related spectrum can be estimated as


(5) $$\begin{array}{r} S\left(f,t\right)=\frac{\frac{1}{n}\sum_{i=1}^{n}|F_{i}\left(f,t\right){|}^{2}}{\mu_{B}^{\prime }\left(f\right)} \end{array}$$

where $\mu_{B}^{\prime }\left(f\right)$ is the mean spectral estimate for all baseline points at frequency *f* and defined as


(6) $$\begin{array}{r} \mu_{B}^{\prime }\left(f\right)=\frac{1}{mn}\overset{n}{\underset{i=1}{\sum }}\underset{t^{\prime }\in B}{\sum }|F_{i}\left(f,t^{\prime }\right){|}^{2} \end{array}$$

where *n* is the number of trials to average the mean calculation. The mean ERSP can be calculated as


(7) $$\begin{array}{r} ERSP_{mean}=10log_{10}\left(S\left(f,t\right)\right) \end{array}$$

#### 3.3.2. Clustered feature

In this study, we suppose that each single trial ERSP is distributed in a high dimensional space. There must be a ground truth value which represents a specific motor imagery existing in this space. The assumption is that all the single trial ERSP is distributed near the ground truth value within a threshold distance. We apply the bootstrap method and randomly pick *k* trials as the center cluster to estimate the ground truth value. The Euclidean distance from single trial to estimated ERSP can be calculated as


(8) $$\begin{array}{r} d_{i}=\|ERSP_{single}-\tilde{ERSP_{i}}{\|}_{2} \end{array}$$

where the *ERSP*_*single*_ is acquired from Equation (4), $\tilde{ERSP_{i}}$ is the estimated ERSP of a specific motor imagery, *i* ∈ {*Griping, Opening, Pronation, Supination*}, $d_{i}\in {\mathbb{R}}^{fb\times 1}$ and *fb* is frequency band. For binary classification such like griping vs. opening or pronation vs. supination, the ERSP distance feature of a channel can be denoted as


(9) $$\begin{array}{r} D_{j}=\left[d_{griping},d_{opening}\right]\;or\;D_{j}=\left[d_{pronation},d_{supination}\right] \end{array}$$

where $D_{j}\in {\mathbb{R}}^{2fb\times 1}$ is the distance feature of *j*th channel. Therefore, the multi-channel feature can be denoted as


(10) $$\begin{array}{r} F=\left[D_{1},D_{2},...,D_{j}\right] \end{array}$$

where *F* ∈ ℝ^2*fb*×*nc*^ and *nc* is the number of channels.

### 3.4. Classification

In this section, we introduce two classical machine learning classifiers such as support vector machine (SVM) and linear discriminate analysis (LDA).

#### 3.4.1. Discriminant analysis models (LDA)

There are various discriminant analysis models such as regularized linear, quadratic etc. Discriminant analysis attempts to maximize the variance between the classes and minimize the variance within the classes by mapping the data to a low dimensional space and assuming the classes' covariance. The prediction function is described as (Malki et al., 2015):


(11) $$\begin{array}{r} \hat{\omega }=\underset{\omega =1,2,...K}{\arg\min}\overset{K}{\underset{k=1}{\sum }}\hat{P}\left(k|x\right)C\left(\omega |k\right) \end{array}$$

where $\hat{\omega }$ is the predicted classification, *K* is the number of classes, $\hat{P}\left(k|x\right)$ is the posterior probability of class *k* for observation *x* and *C*(ω|*x*) is cost of classifying an observation as ω when its true class is *k*. Regularized linear discriminant analysis (LDA) in which all classes have the same covariance matrix is employed for further classification. Meanwhile, we utilize MATLAB 2022 to optimize the hyperparameter automatically and apply “expected-improvement-plus” as optimization options.

#### 3.4.2. Support vector machine (SVM)

In binary classification problems, support vector machine(SVM) shows an effective role. SVM algorithm is to find a hyperplane which can separate two classes with the largest margin by projecting the input vector into a high dimensional space. The hyperplane we expected can separate two classes with the largest margin where margin means the maximum width of the parallel to the hyperplane that has no interior data points. For training data d dimension *x*_*j*_ with their categories *y*_*i*_, the expected hyperplane can be denoted as:


(12) $$\begin{array}{r} \hat{f}\left(x\right)=x^{\prime }\hat{\beta }+\hat{b}=0 \end{array}$$

where $x_{j}\in {\mathbb{R}}^{d}$, *y*_*i*_ = ±1, $\hat{\beta }$ and $\hat{b}$ meet the inequality $y_{i}\hat{f}\left(x_{j}\right)\geq 1$. Therefore, the prediction function can be denoted as:


(13) $$\begin{array}{r} class\left(z\right)=sign\left(z^{\prime }\hat{\beta }+\hat{b}\right)=sign\hat{f}\left(z\right) \end{array}$$

where *z* is the predicted data. We employ a linear kernel to set SVM and utilize the same hyperparameter optimization option as LDA in MATLAB 2022.

## 4. Result

### 4.1. Accuracy based on single trial ERSP estimate

The baseline time is set at when the visual stimulus emerges. A 5,000 ms lasting motor imagery epoch which is from −1,000 ms before baseline to 4,000 ms after baseline is extracted in the ERSP calculation. Ten-fold cross validation is applied on single trial ERSP to train the SVM and LDA. The multi channels which cover the motor cortex contain 21 channels (FC5, FC3, FC1, FCz, FC2, FC4, FC6, C5, C3, C1, Cz, C2, C4, C6, CP5, CP3, CP1, CPz, CP2, CP4, CP6) as Figure 2B. Table 1 shows the average accuracy and corresponding standard deviation of 9 subjects in griping vs. opening classification. Based on single channel ERSP, the best accuracy of the SVM model is 55.0% from subject 9 and the average accuracy is 47.95%. The best accuracy of the LDA model is 59.59% from subject 8, and the average accuracy is 51.28%. The average loss is 0.51 of SVM and 0.48 of LDA. Based on the multi-channel ERSP from motor cortex area, the best accuracy of the SVM model is 55.94% from subject 9 and the average accuracy is 51.15%. The best accuracy of the LDA model is 59.06% from subject 8, and the average accuracy is 52.50%. The average loss is 0.49 of the SVM and 0.48 of the LDA.

**Table 1:** Griping vs. opening classification based on ERSP estimation.

**Subject**	**SVM (%)**		**LDA (%)**	
	**C3 channel (single)**	**Motor cortex (multi)**	**C3 channel (single)**	**Motor cortex (multi)**
S1	49.38 ± 5.67	53.75 ± 5.47	52.81 ± 7.13	56.25 ± 4.66
S2	35.94 ± 6.12	52.81 ± 4.53	51.88 ± 3.95	53.13 ± 5.31
S3	49.38 ± 3.55	35.94 ± 3.97	40.62 ± 2.08	35.31 ± 5.11
S4	48.75 ± 3.95	53.75 ± 5.47	54.69 ± 6.63	57.81 ± 5.36
S5	51.56 ± 5.16	54.38 ± 9.34	48.44 ± 5.75	56.25 ± 4.89
S6	44.69 ± 6.76	50.31 ± 4.02	51.25 ± 5.74	50.63 ± 2.87
S7	44.38 ± 1.32	50.94 ± 5.11	48.75 ± 2.63	49.38 ± 2.57
S8	52.50 ± 5.47	52.50 ± 4.37	**59.69** **±5.79**	**59.06** **±4.84**
S9	**55.00** **±5.35**	**55.94** **±4.98**	53.44 ± 3.74	54.69 ± 3.97
Average	47.95 ± 7.21	51.15 ± 7.69	51.28 ± 6.95	52.50 ± 8.41

*Accuracy (%) and standard deviation (±) for single channel and multi-channels in two classifiers. The best results are boldfaced*.

Similarly, Table 2 shows the performance of SVM and LDA in the pronation vs. supination classification. Based on single channel ERSP, subject 3 obtains the best accuracy in both SVM (62.50%) and LDA (59.06%) models. The average accuracy is 52.19% of SVM and 52.32% of LDA. The average loss is 0.47 of the SVM and 0.48 of the LDA. Based on motor cortex area channels, the best accuracy of the SVM model is 56.25% from subject 6 and the best accuracy of the LDA model is 58.44% from subject 1. The average accuracy is 50.66% of the SVM and 48.13% of the LDA. The average loss is 0.49 of the SVM and 0.52 of the LDA.

**Table 2:** Pronation vs. supination classification based on ERSP estimation.

**Subject**	**SVM (%)**		**LDA (%)**	
	**C3 channel (single)**	**Motor cortex (multi)**	**C3 channel (single)**	**Motor cortex (multi)**
S1	45.63 ± 4.93	53.13 ± 4.17	45.63 ± 4.47	**58.44** **±3.31**
S2	57.81 ± 5.36	46.88 ± 5.89	56.25 ± 3.61	47.50 ± 5.86
S3	**62.50** **±6.42**	52.50 ± 3.84	**59.06** **±3.74**	45.63 ± 7.25
S4	52.50 ± 8.18	41.88 ± 6.46	51.88 ± 5.15	41.88 ± 4.47
S5	52.81 ± 3.44	53.13 ± 4.42	51.56 ± 6.29	50.94 ± 5.71
S6	51.56 ± 3.68	**56.25** **±5.31**	52.19 ± 3.62	56.88 ± 3.55
S7	46.88 ± 7.66	46.88 ± 2.95	46.25 ± 6.39	36.56 ± 5.11
S8	55.94 ± 6.82	53.13 ± 2.55	57.81 ± 6.63	42.81 ± 6.43
S9	43.12 ± 6.38	52.18 ± 2.57	50.31 ± 0.99	52.50 ± 3.54
Average	52.19 ± 8.19	50.66 ± 6.01	52.32 ± 6.43	48.13 ± 8.46

*Accuracy (%) and standard deviation (±) for single channel and multi-channels in two classifiers. The best results are boldfaced*.

### 4.2. Accuracy based on clustered feature

**Figure 4:**
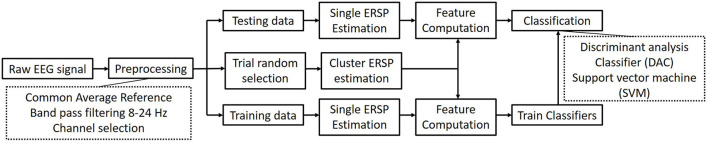
The architecture of the binary right hand motor imagery classification.

In this section, 20 trials are utilized to estimate the mean ERSP value as the center cluster reference. The binary classification strategy is implemented as Figure 4 shown. Table 3 shows the average accuracy and corresponding standard deviation of 9 subjects in griping vs. opening classification. Through applying clustered features on a single channel, the best accuracy is 91.25% of SVM model and 90.94% of LDA model from subject 6. The average accuracy is 76.15% of the SVM and 71.81% of the LDA. The average loss is 0.28 and 0.24, respectively. Through applying multi-channel clustered features, subject 1 obtains the best accuracy (95.94%) of SVM and subject 7 obtains the best accuracy (95.63%) of LDA. The average accuracy is 85.87% of the SVM and 84.03% of the LDA. The average loss is 0.14 and 0.16, respectively.

**Table 3:** Griping vs. opening classification based on clustered features.

**Subject**	**SVM (%)**		**LDA (%)**	
	**C3 channel (single)**	**Motor cortex (multi)**	**C3 channel (single)**	**Motor cortex (multi)**
S1	74.38 ± 2.47	**95.94** **±2.57**	75.31 ± 2.31	92.50 ± 2.64
S2	79.38 ± 3.02	82.50 ± 3.01	79.69 ± 2.21	81.25 ± 4.42
S3	72.50 ± 4.11	90.63 ± 3.90	65.31 ± 6.66	84.69 ± 4.76
S4	78.75 ± 4.37	81.25 ± 4.42	74.69 ± 3.74	81.56 ± 2.31
S5	88.13 ± 3.84	83.13 ± 5.55	73.13 ± 7.68	79.38 ± 5.35
S6	**91.25** **±1.32**	83.75 ± 3.23	**90.94** **±0.99**	83.44 ± 3.62
S7	72.81 ± 2.57	94.69 ± 2.11	72.19 ± 3.44	**95.63** **±2.19**
S8	62.81 ± 4.53	80.31 ± 3.91	51.25 ± 9.57	76.56 ± 6.12
S9	65.31 ± 3.10	80.63 ± 2.87	63.75 ± 3.36	81.25 ± 3.29
Average	76.15 ± 9.51	85.87 ± 6.81	71.81 ± 11.61	84.03 ± 7.03

*Accuracy (%) and standard deviation (±) for single channel and multi-channels in two classifiers. The best results are boldfaced*.

Table 4 shows the average accuracy and corresponding standard deviation of 9 subjects in pronation vs. supination classification. Subject 7 obtains the best accuracy in both single (94.06% of the SVM and 90.94% of the LDA) and multi-channels clustered features (98.75% of SVM and 97.50% of LDA). The average accuracy based on single channel clustered features is 74.89% of the SVM and 72.88% of the LDA. The average loss is 0.27 and 0.25, respectively. Besides, the average accuracy based on multi-channel clustered features is 84.55% of the SVM and 83.81% of the LDA. The average loss is 0.15 and 0.16, respectively.

**Table 4:** Pronation vs. supination classification based on clustered features.

**Subject**	**SVM (%)**		**LDA (%)**	
	**C3 channel (single)**	**Motor cortex (multi)**	**C3 channel (single)**	**Motor cortex (multi)**
S1	74.06 ± 3.62	71.88 ± 3.61	71.25 ± 2.47	74.38 ± 4.61
S2	77.19 ± 2.57	97.19 ± 1.77	78.13 ± 3.90	95.31 ± 2.66
S3	73.44 ± 3.38	84.06 ± 1.77	69.38 ± 3.84	90.00 ± 2.87
S4	81.88 ± 3.84	82.81 ± 5.36	77.19 ± 5.71	77.81 ± 5.20
S5	78.44 ± 3.11	73.44 ± 5.56	74.06 ± 2.96	75.00 ± 5.71
S6	67.50 ± 4.93	94.06 ± 3.44	68.75 ± 4.17	86.25 ± 4.70
S7	**94.06** **±1.77**	**98.75** **±1.61**	**90.94** **±4.53**	**97.50** **±1.32**
S8	65.31 ± 2.31	76.88 ± 5.35	66.88 ± 6.46	78.13 ± 4.42
S9	62.19 ± 1.77	81.88 ± 4.11	59.38 ± 1.47	80.00 ± 7.39
Average	74.89 ± 9.64	84.55 ± 10.22	72.88 ± 9.28	83.81 ± 9.41

*Accuracy (%) and standard deviation (±) for single channel and multi-channels in two classifiers. The best results are boldfaced*.

### 4.3. Parameter tuning

#### 4.3.1. Trial number for clustered feature calculation

In this part, the accuracy of calculating the multi-channel clustered feature by different trial number is enumerated. Table 5 shows the accuracy of SVM model performance when applying clustered feature on multi-channel in griping and opening classification. All the subjects obtain the highest accuracy when utilizing 10 trials to compute the multi-channel clustered feature. The accuracy drops a lot from 90.77 to 84.03% when comparing the result of applying 10 and 20 trials. The accuracy of all subjects drops gradually and slightly when the trial number of calculating multi-channel clustered features raises from 20 to 80. The mean average varies within 5% between adjacent two groups.

**Table 5:** The accuracy (%) for applying different trial numbers to calculate the multi-channels clustered feature in griping vs. opening classification.

**Trial number**	**S1**	**S2**	**S3**	**S4**	**S5**	**S6**	**S7**	**S8**	**S9**	**Average**
10	93.44	92.50	91.56	85.63	90.63	94.06	97.19	88.44	83.44	90.77
20	**92.50**	**81.25**	**84.69**	**81.56**	**79.38**	**83.44**	**95.63**	**76.56**	**81.25**	**84.03**
30	89.69	74.38	82.19	76.25	70.63	80.94	97.50	65.31	89.06	80.66
40	83.44	69.06	65.94	72.81	65.94	76.88	93.44	61.88	90.31	75.52
50	90.31	77.19	71.56	72.81	70.94	75.00	94.38	66.25	76.56	77.22
60	75.31	72.50	62.81	70.31	64.38	69.69	85.00	62.81	79.06	71.31
70	83.44	73.13	66.56	70.00	66.56	63.75	90.31	64.69	75.94	72.70
80	81.88	79.06	64.06	69.69	65.63	72.50	78.44	68.75	80.63	73.41

*The boldfaced accuracy is selected as default trial number*.

#### 4.3.2. Clustered ERSP estimation of different baseline

The key point of event related spectral perturbation is the baseline setting. According to the previous experimental report and the assumption we proposed, an observation period is added to enhance the motor imagery activities in our experiment design. As a result, we utilize a different baseline to calculate the cluster mean ERSP estimation and compare the performance in both classifiers based on the multi-channel clustered feature in this part. Based on the SVM model, onlyăsubject 3 and 8 in the observation period obtain a better accuracy than others in the imagery period. Meanwhile, subject 2 and 8 in the observation period obtained better accuracy than others in the imagery period based on the LDA model. In general, the clustered feature estimated by imagery period baseline yields a better result than observation period baseline. (In SVM, observation vs. imagery, *p* = 0.02, In LDA, observation vs. imagery, *p* = 0.08).

#### 4.3.3. Channel selection

It is necessary to take the channel selection into account. As the description above, 21 channels are employed to calculate the clustered features because these channels cover the brain motor cortex area and record the EEG signals for motor imagery tasks. Considering both contralateral motor cortex and the ipsilateral motor cortex has its own physiological effect on brain activities, we test the clustered features performance by applying different channel selection combinations in this part. As **Table 7** shown, channel FC5, FC3, FC1, C5, C3, C1, CP5, CP3, CP1 represent the left contralateral motor cortex while channel FC6, FC4, FC2, C6, C4, C2, CP6, CP4, CP2 represent the right ipsilateral motor cortex. In the LDA model, full motor cortex channel selection obtains the best accuracy except for subject 6 by single C3 channel, subject 8 by single C4 channel and subject 7,9 by left contralateral motor cortex. In addition, the average accuracy is 72.71% based on the C4 channel, 78.47% based on the left motor cortex, and 77.99% based on the right motor cortex. In addition, only the *p*-value (full motor cortex channels vs. single channel C3 or C4) is <0.05.

#### 4.3.4. Frequency band selection

As the most informative frequency band in motor imagery tasks, μ band (8–12 Hz) and β band (16–24 Hz) play a vital role in the EEG signal analysis. Considering their different physiological meanings, the clustered feature performance of LDA model in each frequency is illustrated as **Table 8** shown. The average accuracy of μ band is 75.31% while the average accuracy of β band is 79.20%. In addition, the wide band (8–24 Hz) which contains the μ band and β band obtains the average accuracy at 84.03%. The *p*-value of wide band vs. μ band is <0.05 while the *p*-value of wide band vs. μ band and β band vs. μ band is >0.05.

## 5. Discussion

### 5.1. Performance of classification

**Figure 5:**
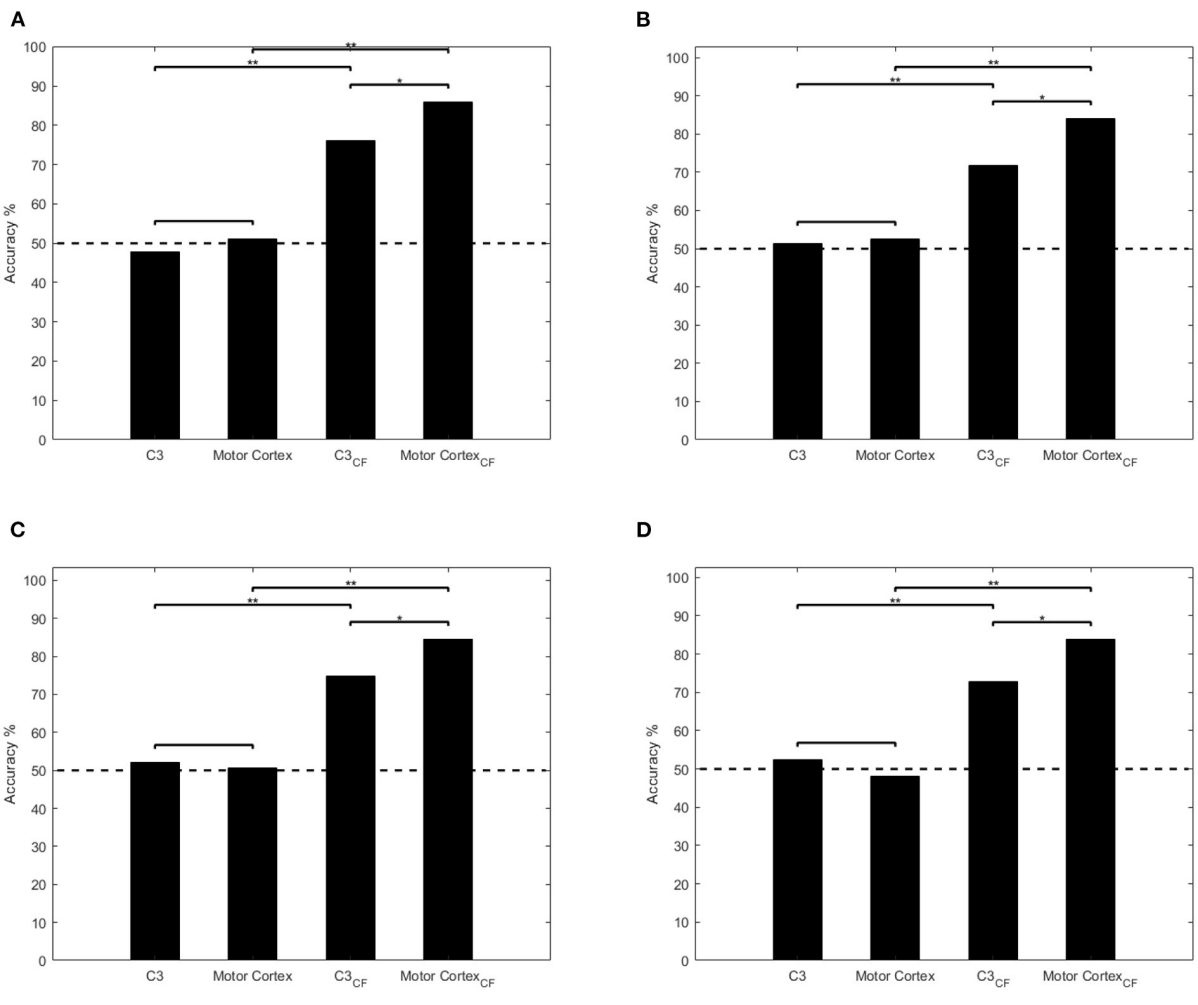
Comparisons of classification performances using single channel ERSP, multi-channel ERSP, single channel clustered feature, and multi-channel feature. *p < 0.05, **p < 0.001. Dashed line denotes the chance level and CF represents clustered feature. **(A,B)** Illustrate the ANOVA test result of SVM and LDA in griping vs. opening classification. **(C,D)** illustrate the ANOVA test result of SVM and LDA in pronation vs. supination classification.

The classification result and significance level is compared as Figure 5 shown. In griping vs. opening classification (Figures 5A,B), the average accuracy (47.95, 51.15, 51.28, and 52.50%) is around chance level (50%) through single channel and multi-channel ERSP input. The result shows the low performance of SVM and LDA which means the classifiers cannot work properly to distinguish these two classes. Through applying clustered feature on single channel and multi-channel, the accuracy raises from 47.95 to 76.15%, 51.15 to 85.87% in SVM model and 51.28 to 71.81%, 52.50 to 84.03% in LDA.

Similarly, in pronation vs. supination classification (Figures 5C,D), both classifiers, whose accuracy are 52.19, 50.66, 52.32, and 48.13%, cannot distinguish the motor imagery through single and multi-channel ERSP input as well. By applying clustered feature on single channel and multi-channel, the accuracy raises from 52.19 to 74.89%, 50.66 to 85.87% in SVM model and 52.32 to 72.88%, 48.13 to 84.03% in LDA.

From statistical analysis as Figure 5 shown, there is a significant improvement by applying clustered feature (*p* < 0.001) in all classifiers and motor imagery classifications. In addition, there also is a significant difference between single channel and multi-channel clustered feature input in both classifiers(SVM and LDA).

### 5.2. Parameter setting

The feature extraction method we proposed in this paper is utilizing a mean ERSP estimation as a cluster to calculate the Euclidean distance. The main factor which highly affects the performance is how many trials are suitable for the cluster ERSP estimation. The result from Table 5 shows that the average accuracy drops gradually with the increasement of trials. However, from the statistical analysis, the *p* < 0.05 between each group except trial number equaling to 10. The result shows that there's no significant difference between utilizing 20 or up to 80 trials to calculate the clustered feature. In the cluster methods, the centroid value may deviate from the true value with the reduction of trial number for estimation. This causes the overfitting during the classifier's training and tends to obtain a very high accuracy. Therefore, there's a trade off in the cluster ERSP estimation. In this paper, we employ 20 trials to estimate the cluster ERSP considering the size of the dataset.

As for baseline setting, the observation period which is supposed to enhance the motor imagery activities causes the baseline changing of the ERSP estimation in our experiment design. The result from Table 6 shows that imagery baseline estimation yields a better accuracy than observation period baseline. However, the *p*-value of two classifiers in both conditions shows that there is no significant difference between two baselines (*p* = 0.02 in the SVM and *p* = 0.08 in the LDA). As a result, we set 1,000 ms before imagery epoch as baseline considering better accuracy and signal continuity.

**Table 6:** The multi-channel clustered feature calculation based on different baseline time in griping vs. opening classification.

**Subject**	**SVM (%)**		**LDA (%)**	
	**Observation baseline**	**Imagery baseline**	**Observation baseline**	**Imagery baseline**
S1	76.88 ± 4.22	**95.94** **±2.57**	78.44 ± 4.02	**92.50** **±2.64**
S2	78.44 ± 3.44	**82.50** **±3.01**	**81.25** **±2.94**	81.25 ± 4.42
S3	75.63 ± 2.46	**90.63** **±3.90**	76.56 ± 3.37	**84.69** **±4.76**
S4	72.19 ± 2.73	**81.25** **±4.42**	74.69 ± 5.19	**81.56** **±2.31**
S5	72.50 ± 5.06	**83.13** **±5.55**	72.50 ± 2.87	**79.38** **±5.35**
S6	**90.00** **±3.23**	83.75 ± 3.23	**90.00** **±2.47**	83.44 ± 3.62
S7	87.81 ± 1.77	**97.19** **±0.99**	94.69 ± 2.11	**95.63** **±2.19**
S8	75.31 ± 3.74	**80.31** **±3.91**	68.75 ± 4.66	**76.56** **±6.12**
S9	80.00 ± 1.61	**80.63** **±2.87**	75.94 ± 3.62	**81.25** **±3.29**
Average	78.40 ± 6.96	**85.87** **±6.81**	78.40 ± 7.34	**84.03** **±7.03**

*The better accuracy (%) and standard deviation (±) are boldfaced*.

In addition, we select different channels and frequency bands to explore the best combination in the motor imagery task. The accuracy from Table 7 illustrates that the channels covering the full motor cortex area obtains the best result. Based on ANOVA test, single contralateral channel C3 shows no significant difference with the single ipsilateral channel C4. Similarly, there is no significant difference between multi contralateral channels (i.e., left motor cortex), multi ipsilateral channels (i.e., right motor cortex), and full motor cortex channels (i.e., 21 channels). However, clustered features based on the full motor cortex area have significant differences with single channel C3 and C4. Therefore, we employ 21 channels to compute the clustered feature considering its better accuracy. The accuracy from Table 8 compares the LDA performance based on multi- channel clustered feature different frequency bands. The wide frequency band obtains the best average accuracy though subject 8 obtains the best accuracy in μ band and subject 6 obtains the best accuracy in β band. The ANOVA test shows that the wide frequency band feature has the significant difference with the μ band feature while no significant difference with the β band feature. As a result, the wide frequency band is extracted to compute the clustered feature in our classification strategy.

**Table 7:** The LDA model performance of clustered feature based on different channel selection in griping vs. opening classification.

	**C3 channel**	**C4 channel**	**Left cortex**	**Right cortex**	**Motor cortex**
S1	75.31 ± 2.31	80.00 ± 4.21	94.69 ± 1.51	84.06 ± 1.77	**92.50** **±2.64**
S2	79.69 ± 2.21	70.94 ± 3.62	78.75 ± 5.06	74.06 ± 3.31	**81.25** **±4.42**
S3	65.31 ± 6.66	67.19 ± 3.68	61.88 ± 4.11	78.13 ± 2.08	**84.69** **±4.76**
S4	74.69 ± 3.74	72.50 ± 3.84	74.69 ± 5.19	73.75 ± 3.67	**81.56** **±2.31**
S5	73.13 ± 7.68	67.19 ± 5.16	76.25 ± 4.47	71.88 ± 3.90	**79.38** **±5.35**
S6	**90.94** **±0.99**	85.63 ± 3.02	83.75 ± 2.87	85.94 ± 3.04	83.44 ± 3.62
S7	72.19 ± 3.44	75.94 ± 2.11	**99.06** **±1.51**	95.94 ± 3.31	95.63 ± 2.19
S8	51.25 ± 9.57	**76.88** **±2.82**	55.94 ± 4.53	74.69 ± 3.44	76.56 ± 6.12
S9	63.75 ± 3.36	58.13 ± 3.95	**81.25** **±2.09**	63.44 ± 4.90	81.25 ± 3.29
Average	71.81 ± 11.61	72.71 ± 8.43	78.47 ± 13.57	77.99 ± 9.53	**84.03** **±7.03**

*The best accuracy (%) and standard deviation (±) are boldfaced*.

**Table 8:** The LDA model performance of clustered feature based on different frequency band in griping vs. opening classification.

	**S1**	**S2**	**S3**	**S4**	**S5**	**S6**	**S7**	**S8**	**S9**	**Average**
μ band (8–12 Hz)	90.94	61.25	70.94	**82.81**	53.75	79.06	91.56	68.75	78.75	75.31
β band (16–24 Hz)	74.69	78.75	77.19	80.00	76.87	**90.31**	**96.88**	67.81	70.31	79.20
Wide band (8–24 Hz)	**92.50**	**81.25**	**84.49**	81.56	**79.38**	83.44	95.63	**76.56**	**81.25**	**84.03**

*The best accuracy (%) are boldfaced*.

### 5.3. Comparative result

In motor imagery research based on EEG signals, feature extraction algorithms such as common spatial pattern (CSP) and filter bank common spatial pattern (FBCSP) are widely employed. Suwannarat et al. (2018) utilized these methods on same hand motor imagery classification. Meanwhile, Chu et al. (2020) applied tangent space (TS) features and partial least square (PLS) algorithms to improve the performance of discriminant analysis classifiers. We compare our proposed feature with their results of the same classifier(LDA) and show the accuracy as Table 9 follows.

**Table 9:** Comparative accuracy (%) with other features in linear discriminant analysis.

**Motor imagery classification**	**CSP**	**FBCSP**	**TS**	**TS-PLS**	**CF**
Griping vs. Opening	61.90	66.19	71.18	80.02	**84.03**
Pronation vs. Supination	66.38	71.69	66.58	78.55	**83.81**

*The result of our method (clustered feature, CF) is boldfaced*.

Clustered feature yields a better accuracy than applying CSP, FBCSP and tangent feature while has the similar accuracy to TS-PLS (80.02% in griping vs. opening and 78.55% in pronation vs. supination). However, our proposed feature also has limitations that it needs prior information to estimate the cluster.

## 6. Conclusion

In this paper, we estimate the mean ERSP from EEG signal. Then we employ the mean ERSP as cluster reference to calculate the feature by Euclidean distance and utilize the clustered feature to train two classical machine learning classifiers. The accuracy obtained by both classifiers in all motor imagery tasks is over 80%. After fast configuration on the subject to estimate the clustered feature, we propose a possibility to apply on online analysis.

In future work, we will consider the optimization method on cluster estimation, develop an adaptive method on searching the best channels and employ other feature extraction algorithms. On the basis of this paper, we will develop a real-time brain computer interface such as robotic grasping system, prosthesis controlling, etc.

## Data availability statement

The raw data supporting the conclusions of this article will be made available by the authors, without undue reservation.

## Ethics statement

The studies involving human participants were reviewed and approved by Ethics Committees of the Tokyo Institute of Technology (Ethics Number: 2019001). The patients/participants provided their written informed consent to participate in this study.

## Author contributions

ZZ and YK contributed to conception and design of the study. ZZ organized the database, performed the statistical analysis, wrote the first draft, and sections of the manuscript. Both authors contributed to manuscript revision, read, and approved the submitted version.

## Conflict of interest

The authors declare that the research was conducted in the absence of any commercial or financial relationships that could be construed as a potential conflict of interest.

## Publisher's note

All claims expressed in this article are solely those of the authors and do not necessarily represent those of their affiliated organizations, or those of the publisher, the editors and the reviewers. Any product that may be evaluated in this article, or claim that may be made by its manufacturer, is not guaranteed or endorsed by the publisher.

## References

[B1] Abbas W. Khan N. A. (2018). FBCSP-based multi-class motor imagery classification using BP and TDP features, in 2018 40th Annual International Conference of the IEEE Engineering in Medicine and Biology Society (EMBC) (Honolulu, HI), 215–218. 10.1109/EMBC.2018.8512238 30440376

[B2] Adeli H. Zhou Z. Dadmehr N. (2003). Analysis of EEG records in an epileptic patient using wavelet transform. J. Neurosci. Methods 123, 69–87. 10.1016/S0165-0270(02)00340-0 12581851

[B3] Akin M. (2002). Comparison of wavelet transform and FFT methods in the analysis of EEG signals. J. Med. Syst. 26, 241–247. 10.1023/A:1015075101937 12018610

[B4] Ang K. K. Chin Z. Y. Wang C. Guan C. Zhang H. (2012). Filter bank common spatial pattern algorithm on BCI competition IV datasets 2a and 2b. Front. Neurosci. 6, 39. 10.3389/fnins.2012.00039 22479236 PMC3314883

[B5] Bashashati A. Fatourechi M. Ward R. K. Birch G. E. (2007). A survey of signal processing algorithms in brain-computer interfaces based on electrical brain signals. J. Neural Eng. 4, R32. 10.1088/1741-2560/4/2/R03 17409474

[B6] Bentlemsan M. Zemouri E.-T. Bouchaffra D. Yahya-Zoubir B. Ferroudji K. (2014). Random forest and filter bank common spatial patterns for EEG-based motor imagery classification, in 2014 5th International Conference on Intelligent Systems, Modelling and Simulation (Langkawi), 235–238. 10.1109/ISMS.2014.46

[B7] Chu Y. Zhao X. Zou Y. Xu W. Song G. Han J.. (2020). Decoding multiclass motor imagery EEG from the same upper limb by combining Riemannian geometry features and partial least squares regression. J. Neural Eng. 17, 046029. 10.1088/1741-2552/aba7cd 32780720

[B8] Corley I. A. Huang Y. (2018). Deep EEG super-resolution: upsampling EEG spatial resolution with generative adversarial networks, in 2018 IEEE EMBS International Conference on Biomedical & Health Informatics (BHI) (Las Vegas, NV), 100–103. 10.1109/BHI.2018.8333379

[B9] da Silva F. L. (1991). Neural mechanisms underlying brain waves: from neural membranes to networks. Electroencephalogr. Clin. Neurophysiol. 79, 81–93. 10.1016/0013-4694(91)90044-5 1713832

[B10] Das R. Lopez P. S. Khan M. A. Iversen H. K. Puthusserypady S. (2020). FBCSP and adaptive boosting for multiclass motor imagery BCI data classification: a machine learning approach, in 2020 IEEE International Conference on Systems, Man, and Cybernetics (SMC) (Toronto, ON), 1275–1279.

[B11] Decety J. Ingvar D. H. (1990). Brain structures participating in mental simulation of motor behavior: a neuropsychological interpretation. Acta Psychol. 73, 13–34. 10.1016/0001-6918(90)90056-L 2180254

[B12] Elstob D. Secco E. L. (2016). A low cost EEG based BCI prosthetic using motor imagery. arXiv preprint arXiv:1603.02869. 10.5121/ijitcs.2016.6103

[B13] Grandchamp R. Delorme A. (2011). Single-trial normalization for event-related spectral decomposition reduces sensitivity to noisy trials. Front. Psychol. 2, 236. 10.3389/fpsyg.2011.00236 21994498 PMC3183439

[B14] Hjorth B. (1975). An on-line transformation of EEG scalp potentials into orthogonal source derivations. Electroencephalogr. Clin. Neurophysiol. 39, 526–530. 10.1016/0013-4694(75)90056-5 52448

[B15] Hsu W.-Y. Sun Y.-N. (2009). EEG-based motor imagery analysis using weighted wavelet transform features. J. Neurosci. Methods 176, 310–318. 10.1016/j.jneumeth.2008.09.014 18848844

[B16] Kappes H. B. Morewedge C. K. (2016). Mental simulation as substitute for experience. Soc. Pers. Psychol. Compass 10, 405–420. 10.1111/spc3.12257

[B17] Kos' Myna N. Tarpin-Bernard F. Rivet B. (2014). Bidirectional feedback in motor imagery BCIs: learn to control a drone within 5 minutes, in CHI'14 Extended Abstracts on Human Factors in Computing Systems (New York, NY), 479–482. 10.1145/2559206.2574820

[B18] Lawhern V. J. Solon A. J. Waytowich N. R. Gordon S. M. Hung C. P. Lance B. J. (2018). EEGNet: a compact convolutional neural network for EEG-based brain-computer interfaces. J. Neural Eng. 15, 056013. 10.1088/1741-2552/aace8c 29932424

[B19] Lotte F. Congedo M. Lécuyer A. Lamarche F. Arnaldi B. (2007). A review of classification algorithms for EEG-based brain-computer interfaces. J. Neural Eng. 4, R1–R13. 10.1088/1741-2560/4/2/R01 17409472

[B20] Luck S. J. (2014). An Introduction to the Event-Related Potential Technique. MIT Press.

[B21] Ma X. Qiu S. Wei W. Wang S. He H. (2019). Deep channel-correlation network for motor imagery decoding from the same limb. IEEE Trans. Neural Syst. Rehabil. Eng. 28, 297–306. 10.1109/TNSRE.2019.2953121 31725383

[B22] Malki A. Yang C. Wang N. Li Z. (2015). Mind guided motion control of robot manipulator using EEG signals, in 2015 5th International Conference on Information Science and Technology (ICIST) (Changsha), 553–558. 10.1109/ICIST.2015.7289033

[B23] McFarland D. J. McCane L. M. David S. V. Wolpaw J. R. (1997). Spatial filter selection for EEG-based communication. Electroencephalogr. Clin. Neurophysiol. 103, 386–394. 10.1016/S0013-4694(97)00022-2 9305287

[B24] Meng J. Zhang S. Bekyo A. Olsoe J. Baxter B. He B. (2016). Noninvasive electroencephalogram based control of a robotic arm for reach and grasp tasks. Sci. Rep. 6, 1–15. 10.1038/srep38565 27966546 PMC5155290

[B25] Miladinović A. Ajčević M. Jarmolowska J. Marusic U. Silveri G. Battaglini P. P.. (2020). Performance of EEG motor-imagery based spatial filtering methods: a BCI study on stroke patients. Proc. Comput. Sci. 176, 2840–2848. 10.1016/j.procs.2020.09.270

[B26] Neuper C. Scherer R. Reiner M. Pfurtscheller G. (2005). Imagery of motor actions: differential effects of kinesthetic and visual-motor mode of imagery in single-trial EEG. Cogn. Brain Res. 25, 668–677. 10.1016/j.cogbrainres.2005.08.014 16236487

[B27] Perrin F. Pernier J. Bertrand O. Echallier J. F. (1989). Spherical splines for scalp potential and current density mapping. Electroencephalogr. Clin. Neurophysiol. 72, 184–187. 10.1016/0013-4694(89)90180-6 2464490

[B28] Pfurtscheller G. (1992). Event-related synchronization (ERS): an electrophysiological correlate of cortical areas at rest. Electroencephalogr. Clin. Neurophysiol. 83, 62–69. 10.1016/0013-4694(92)90133-3 1376667

[B29] Pfurtscheller G. Aranibar A. (1977). Event-related cortical desynchronization detected by power measurements of scalp EEG. Electroencephalogr. Clin. Neurophysiol. 42, 817–826. 10.1016/0013-4694(77)90235-8 67933

[B30] Pfurtscheller G. Brunner C. Schlögl A. Da Silva F. L. (2006). Mu rhythm (de) synchronization and EEG single-trial classification of different motor imagery tasks. NeuroImage 31, 153–159. 10.1016/j.neuroimage.2005.12.003 16443377

[B31] Pfurtscheller G. Neuper C. (1997). Motor imagery activates primary sensorimotor area in humans. Neurosci. Lett. 239, 65–68. 10.1016/S0304-3940(97)00889-6 9469657

[B32] Polat K. Güneş S. (2007). Classification of epileptiform EEG using a hybrid system based on decision tree classifier and fast Fourier transform. Appl. Math. Comput. 187, 1017–1026. 10.1016/j.amc.2006.09.022

[B33] Ramadan R. A. Vasilakos A. V. (2017). Brain computer interface: control signals review. Neurocomputing 223, 26–44. 10.1016/j.neucom.2016.10.024

[B34] Rodríguez-Ugarte M. Iáñez E. Ortiz M. Azorín J. M. (2018). Improving real-time lower limb motor imagery detection using TDCS and an exoskeleton. Front. Neurosci. 12, 757. 10.3389/fnins.2018.00757 30405340 PMC6206210

[B35] Schirrmeister R. T. Springenberg J. T. Fiederer L. D. J. Glasstetter M. Eggensperger K. Tangermann M.. (2017). Deep learning with convolutional neural networks for EEG decoding and visualization. Hum. Brain Mapp. 38, 5391–5420. 10.1002/hbm.23730 28782865 PMC5655781

[B36] Srinivasan R. (1999). Methods to improve the spatial resolution of EEG. Int. J. Bioelectromagn. 1, 102–111.

[B37] Suwannarat A. Pan-Ngum S. Israsena P. (2018). Comparison of EEG measurement of upper limb movement in motor imagery training system. Biomed. Eng. Online 17, 1–22. 10.1186/s12938-018-0534-0 30071853 PMC6071373

[B38] Tariq M. Trivailo P. M. Simic M. (2019). Classification of left and right knee extension motor imagery using common spatial pattern for BCI applications. Proc. Comput. Sci. 159, 2598–2606. 10.1016/j.procs.2019.09.256

[B39] Togha M. M. Salehi M. R. Abiri E. (2019). Improving the performance of the motor imagery-based brain-computer interfaces using local activities estimation. Biomed. Signal Process. Control 50, 52–61. 10.1016/j.bspc.2019.01.008

[B40] Wang Y. Gao S. Gao X. (2006). Common spatial pattern method for channel selection in motor imagery based brain-computer interface, in 2005 IEEE Engineering in Medicine and Biology 27th Annual Conference (Shanghai), 5392–5395. 10.1109/IEMBS.2005.1615701 17281471

[B41] Yoshimura N. Tsuda H. Kawase T. Kambara H. Koike Y. (2017). Decoding finger movement in humans using synergy of EEG cortical current signals. Sci. Rep. 7, 1–11. 10.1038/s41598-017-09770-5 28900188 PMC5595824

